# Transcriptomic immunologic signature associated with favorable clinical outcome in basal-like breast tumors

**DOI:** 10.1371/journal.pone.0175128

**Published:** 2017-05-04

**Authors:** Sandra Martínez-Canales, Francisco Cifuentes, Miguel López De Rodas Gregorio, Leticia Serrano-Oviedo, Eva María Galán-Moya, Eitan Amir, Atanasio Pandiella, Balázs Győrffy, Alberto Ocaña

**Affiliations:** 1 Translational Research Unit, Albacete University Hospital and CIBERONC, Albacete, Spain; 2 Centro Regional de Investigaciones Biomédicas (CRIB), Universidad de Castilla La Mancha, Albacete, Spain; 3 Princess Margaret Cancer Center, University of Toronto, Toronto, Canada; 4 Cancer Research Center and CIBERONC, CSIC-University of Salamanca, Salamanca, Spain; 5 MTA TTK Lendület Cancer Biomarker Research Group, Budapest, Hungary; 6 Semmelweis University 2nd Dept. of Pediatrics, Budapest, Hungary; Hospital Infantil Universitario Nino Jesus, SPAIN

## Abstract

**Background:**

Most patients with early stage triple negative breast cancer (TNBC) receive adjuvant chemotherapy. Activation of the immune system is associated with tumor response and may help identify TNBC with favorable outcome.

**Methods:**

Gene expression data were obtained from the GEO Dataset GDS2250/GSE3744. Affymetrix CEL files were downloaded and analyzed with Affymetrix Transcriptome Analysis Console 3.0. Functional genomics was implemented with David Bioinformatics Resources 6.8. Data contained at Oncomine were used to identify genes upregulated in basal-like cancer compared to normal breast tissue. Data contained at cBioportal were used to assess for molecular alterations. The KMPlotter online tool, METABRIC and GSE25066 datasets were used to associate gene signatures with clinical outcome.

**Results:**

1564 upregulated genes were identified as differentially expressed between normal and basal-like tumors. Of these, 16 genes associated with immune function were linked with clinical outcome. HLA-C, HLA-F, HLA-G and TIGIT were associated with both improved relapse-free survival (RFS) and overall survival (OS). The combination of HLA-F/TIGIT and HLA-C/HLA-F/TIGIT showed the most favorable outcome (HR for RFS 0.44, p<0.001; HR for OS 0.22, p<0.001; and HR for RFS 0.46, p<0.001; HR for OS 0.15, p<0.001; respectively). The association of HLA-C/HLA-F with outcome was confirmed using the METABRIC and GSE25066 datasets. No copy number alterations of these genes were identified.

**Conclusion:**

We describe a gene signature associated with immune function and favorable outcome in basal-like breast cancer. Incorporation of this signature in prospective studies may help to stratify risk of early stage TNBC.

## Introduction

Metastatic triple negative breast cancer (TNBC) is an incurable disease [1]. However, in early stage disease, surgical resection and adjuvant treatment with chemotherapy can contribute to cure in a substantial proportion of patients [1].

Decisions for recommendation for adjuvant treatment in breast cancer are based on the overall risk of cancer recurrence which can be estimated based on the extent of disease, as well as biological factors such as histologic grade and receptor status. For TNBC given the high risk of relapse and the poor prognosis, treatment with chemotherapy is offered to most patients. However, in some circumstances the benefit from adjuvant chemotherapy in TNBC can be less clear, for instance when tumors are very small, when patients are older or when medical co-morbidities make the toxicity of chemotherapy less favorable [2]. In these circumstances it would be desirable to have better information about prognosis.

Inhibition of immune surveillance is one of the hallmarks of cancer [3]. Activation of immune system against the tumor can lead to an anti-tumor response and therefore potentially improve clinical outcome. In TNBC the presence of tumor infiltrating lymphocytes (TIL) is a marker of the activation of the immune system and is associated with improved outcome [4, 5]. Indeed, therapeutic strategies to activate the immune system, like those blocking the programmed death receptor-1 (PD-1) or its ligand; or cytotoxic T-lymphocyte associated protein 4 (CTLA-4) receptors, have shown activity in several solid tumors [6]. In TNBC these agents are currently in late stages of development [7].

Different cell membrane proteins are associated with the identification of neoantigens and activation of effector T-cells, or the regulation of this activation [8]. This has been the case for antibodies against PD1 and CTLA4, among others [8]. However, many other cell surface regulators involved in this activation exist and have not been explored. An example includes the family of Human Leucocyte Antigens (HLA), or the novel regulatory protein T cell immunoreceptor with Ig and ITIM domains (TIGIT) [9].

In our study, by using an *in silico* approach, we aimed to identify genes that code for cell surface proteins and then select those involved in the regulation of the immune system for further evaluation. We subsequently examined their prognostic influence to help identify patients at lower risk of relapse and for whom treatment with chemotherapy may not be needed.

## Material and methods

### Transcriptomic and gene-set enrichment analyses

We used a public dataset (GEO DataSet accession number: GDS2250) [10] of mRNA level data from normal breast tissue and basal-like breast tumors to identify upregulated genes. Affymetrix CEL files were downloaded and analyzed with Affymetrix Transcriptome Analysis Console 3.0. We obtained genes with different expression values from the two samples and unregulated genes were defined as those with a minimum 2 fold differential expression between normal and malignant tissue. We selected this cut-off level to include a large number of genes. The list of genes was analyzed using gene-set enrichment analyses using DAVID Bioinformatics Resources 6.8 (https://david.ncifcrf.gov/) in order to identify functions of these genes. We used an adjusted p-value of less than 0.05 to select the enriched gene sets. Data contained at Oncomine (www.oncomine.org) was used to independently confirm the difference among the selected genes.

### Outcome analyses

The KM Plotter Online Tool was used to analyze the relationship between the gene expression and patient clinical outcome in breast cancer (http://www.kmplot.com); this public database allowed us to investigate overall survival (OS) and relapse-free survival (RFS) [11]. Definitions of breast cancer subgroups reported in this online tool are: triple negative (ER-/HER2-), luminal A (ESR1+/HER2-/MK167-low), luminal B (ESR1+/HER2-/MK167-high or ESR1+/HER2+), and basal-like (ESR1-/HER2-) [12]. Analysis was carried out in multiple steps. First, we restricted analysis to the basal-like breast cancer subgroup. Then, the association between the genes of interest and outcome was explored after selection of the specific probe utilized initially. Finally, we evaluated all immunologic-related genes and their association with both RFS and OS in basal like breast cancer, and confirmed these data in the Oncomine dataset.

We also confirmed the results using two additional datasets: METABRIC [13] and the GSE25066 dataset [14]. METABRIC contains information about 331 basal-like tumors with data available for OS, while the GSE25066 dataset comprises RFS data on 142 basal-like tumors.

### Evaluation of molecular alterations

To explore the presence of mutations, deletions or amplifications in the identified genes we used data contained at cBioportal (www.cbioportal.org) [15].

## Results

### Identification of upregulated genes coding for membrane cell surface proteins

**Fig 1:**
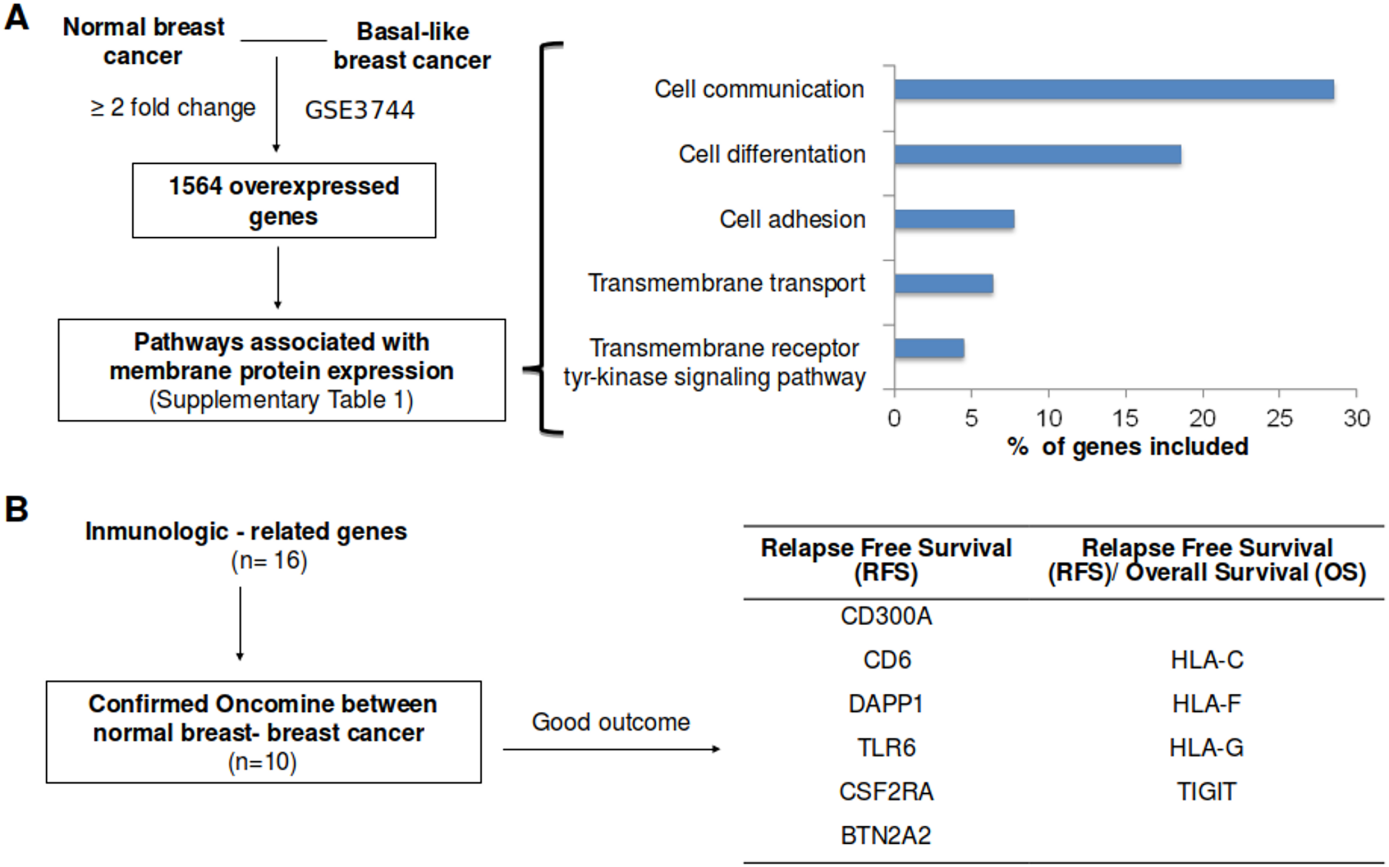
Gene-set enrichment analyses of differentially expressed genes between normal breast and basal like breast cancer, and association with clinical outcome. **A.** Overexpressed genes with a minimum of two fold-change difference between normal breast and basal-like breast cancer (left side), as described in material and methods. Using functional annotation, only genes associated with functions linked with cell membrane proteins were included (right side). **B.** Identification of immunologic related genes included in the identified functions (16 genes). Selection of genes which upregulation was confirmed using data contained at Oncomine (10 genes) and linked with outcome. Outcome screening analyses for RFS and OS using the KMplotter online tool for those ten identified genes.

To identify upregulated genes in TNBC we analyzed data from a public GEO Dataset (accession number: GDS2250/GSE3744) [10]. Using a minimum fold change of 2, we selected 1564 upregulated genes. We evaluated these genes using functional genomics with David Bioinformatics Resources 6.8 (https://david.ncifcrf.gov/). As genes that code for proteins that are at the plasma membrane can be involved in several functions we extracted different functions including cell differentiation, communication, adhesion, transmembrane transport and transmembrane receptor tyrosine kinase signaling pathway (Fig 1A). A total of 934 genes were included in these functions (S1 Table).

### Selection of immune genes linked with outcome

Using information from genes that code for proteins at the cell membrane, we explored only those involved in immunological processes that are associated with outcome, and confirmed their upregulated expression in breast cancer using data available at Oncomine (Fig 1B and S2 Table). A total of 16 genes were identified, but only ten confirmed as upregulated using data from Oncomine and linked with outcome. Using the online tool KM plotter [11] and making the analyses for those ten genes we observed that, CD300A, CD6, DAPP1, TLR6, CSF2RA and BTN2A2 were associated with improved RFS only, while HLA-C, HLA-F, HLA-G and TIGIT were associated with both improved RFS and OS in basal breast cancer patients (Fig 1B).

### Gene set combinations for HLA-C, HLA-F, HLA-G and TIGIT

**Fig 2:**
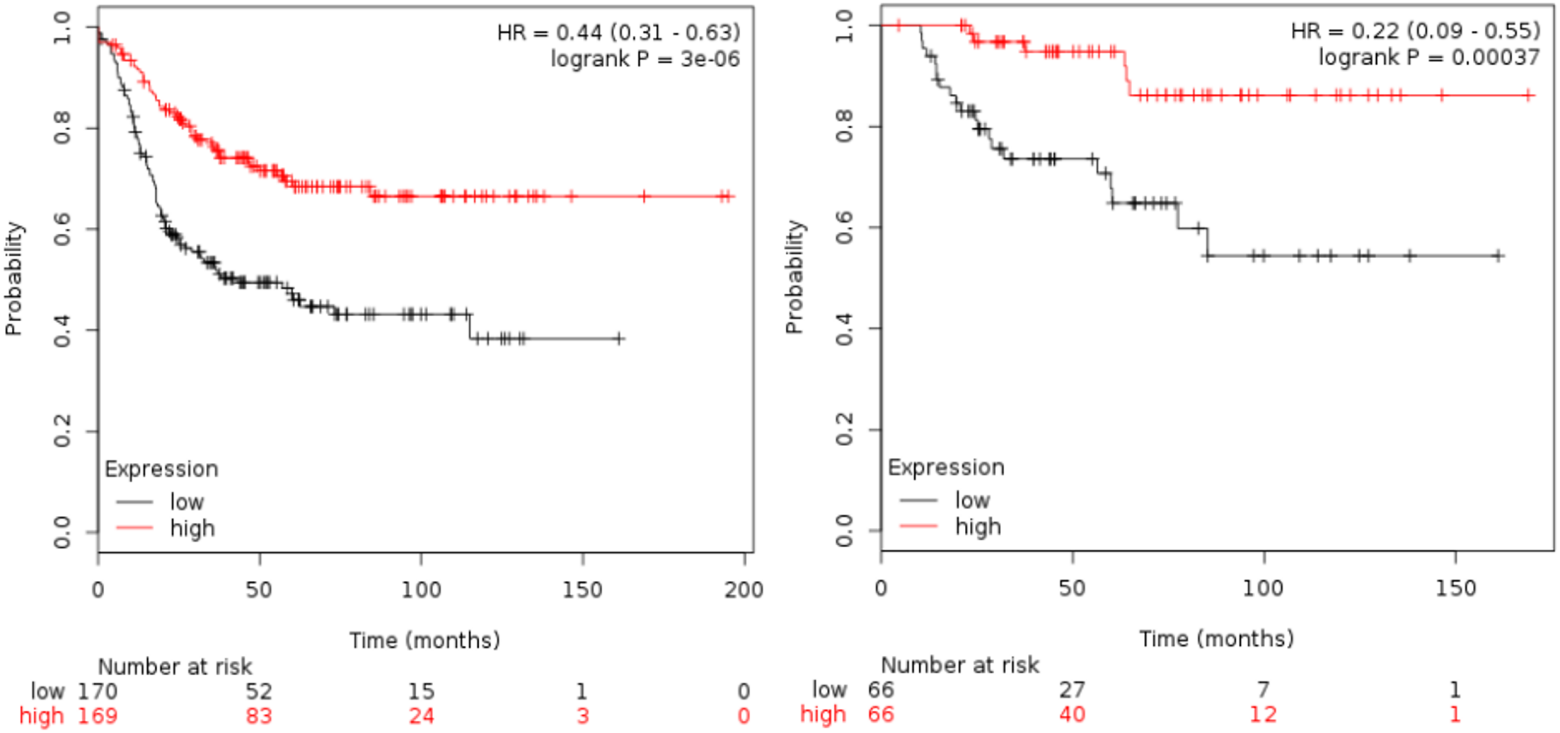
Association of the combined analyses of HLA-F and TIGIT with RFS and OS in basal-like patients using the KM plotter online tool, as described in material and methods.

**Fig 3:**
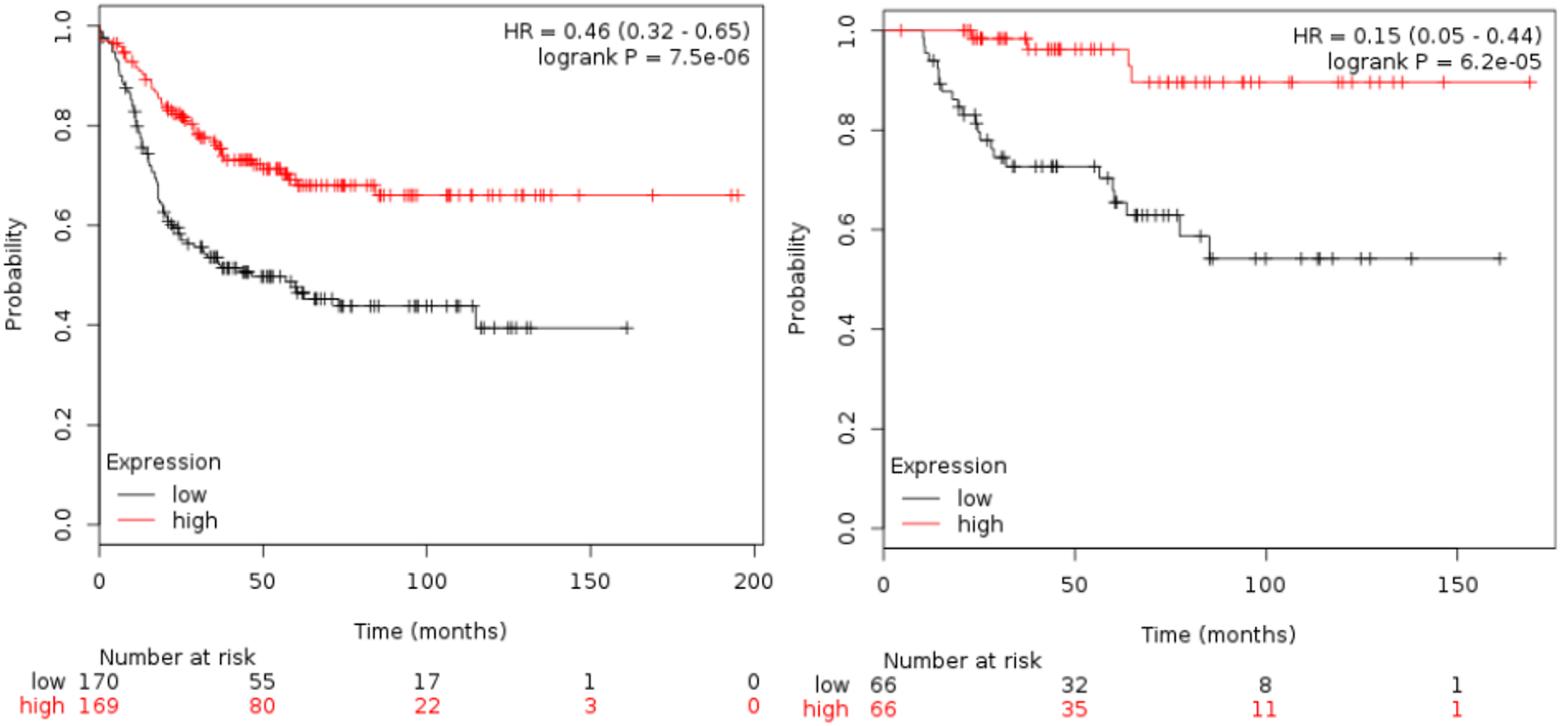
Association of the combined analyses of HLA-F, HLA-C and TIGIT with RFS and OS in basal-like patients using the KM plotter online tool, as described in material and methods.

Table 1 shows the association of all the immunologic-related genes with RFS and OS. TIGIT was the gene associated with the best outcome (HR 0.36 [95% CI 0.25–0.52]; p<0.001 for RFS and HR 0.37 [95% CI 0.16–0.84]; p = 0.01 for OS). Table 2 shows results for the combinations of HLA-C, HLA-F, HLA-G and TIGIT as predictors of patient outcome. The combination of HLA-F and TIGIT, and HLA-C, HLA-F and TIGIT were the two best combinations for clinical outcome. HLA-F and TIGIT (HR for RFS 0.44 [95% CI 0.31–0.63] p<0.001 and HR for OS 0.22 [95% CI 0.09–0.55) p<0.001, see Fig 2). For HLA-C, HLA-F and TIGIT, the HR for RFS was 0.46 [95% CI 0.32–0.65] p<0.001 and HR for OS 0.15 [95% CI 0,05–0,44] p<0.001, see Fig 3). Although the analysis was done using the median, the correlation between survival and the combined gene signature was independent of the used cutoff value for both RFS and OS (S1 Fig).

**Table 1:** List of the identified immunologic-related genes associated with RFS and OS in basal like breast cancer.

Probe Set	Gene name	Basal Like			
		Relapse Free Survival		Overall Survival	
		HR (Hazard Ratio)	Logrank P-value	HR (Hazard Ratio)	Logrank P-value
217072_at	CD300A, CD300a molecule	0,74 (0,57–0,96)	0,023	-	>0,05
211893_x_at	CD6, CD6 molecule	0,53 (0,4–0,69)	2,20E-06	-	>0,05
222859_s_at	DAPP1, dual adaptor of phosphotyrosine and 3-phosphoinositides	0,5 (0,35–0,7)	5,50E-05	-	>0,05
211799_x_at	HLA-C, major histocompatibility complex, class I, C	0,59 (0,45–0,77)	9,90E-05	0,38 (0,21–0,71)	0,0014
221875_x_at	HLA-F, major histocompatibility complex, class I, F	0,53 (0,41–0,7)	2,80E-06	0,35 (0,19–0,63)	0,00032
211528_x_at	HLA-G, major histocompatibility complex, class I, G	0,6 (0,46–0,79)	0,00017	0,42 (0,23–0,75)	0,0028
239021_at	TLR6, toll-like receptor 6	0,67 (0,48–0,95)	0,023	-	>0,05
210340_s_at	CSF2RA, colony stimulating factor 2 receptor, alpha, low-affinity (granulocyte-macrophage)	0,59 (0,45–0,76)	6,80E-05	-	>0,05
240070_at	TIGIT, T cell immunoreceptor with Ig and ITIM domains	0,36 (0,25–0,52)	8,30E-09	0,37 (0,16–0,84)	0,014
1564684_at	BTN2A2, butyrophilin, subfamily 2, member A2	0,55 (0,39–0,78)	0,00072	-	>0,05

**Table 2:** List of gene combinations associated with favorable outcome in basal like cancer using the KM plotter online tool, as described in material and methods.

Gene combinations	Relapse Free Survival			Overall Survival		
	HR (Hazard Ratio)	Logrank P- value	N (patients)	HR (Hazard Ratio)	Logrank P- value	N (patients)
**HLA-C, HLA-F, HLA-G, TIGIT**	0,54 (0,38–0,77)	0.00047	339	0,2 (0,08–0,54)	0,00036	132
**HLA-C, HLA-F, HLA-G**	0,55 (0,42–0,72)	7,8E-06	580	0,38 (0,21–0,7)	0,0011	204
**HLA-C, HLA-F, TIGIT**	0,46 (0,32–0,65)	7,5E-06	339	0,15 (0,05–0,44)	6,2E-05	132
**HLA-C, HLA-G, TIGIT**	0,56 (0,4–0,79)	0,00081	339	0,15 (0,05–0,44)	6,7E-05	132
**HLA-F, HLA-G, TIGIT**	0,5 (0,35–0,71)	7,8E-05	339	0,25 (0,1–0,62)	0,0012	132
**HLA-C, HLA-F**	0,51 (0,39–0,67)	6,2E-07	580	0,31 (0,16–0,58)	0,00011	204
**HLA-C, HLA-G**	0,62 (0,47–0,8)	0,00029	580	0,39 (0,21–0,71)	0,0014	204
**HLA-C, TIGIT**	0,58 (0,41–0,81)	0,0015	339	0,21 (0,08–0,57)	0,00063	132
**HLA-F, HLA-G**	0,57 (0,44–0,75)	3,5E-05	580	0,42 (0,23–0,76)	0,0029	204
**HLA-F, TIGIT**	0,44 (0,31–0,63)	3E-06	339	0,22 (0,09–0,55)	0,00037	132
**HLA-G, TIGIT**	0,57 (0,4–0,81)	0,0013	339	0,19 (0,07–0,51)	2E-04	132

HLA-C, HLA-F, HLA-G and TIGIT were not associated with mutations or copy number gains (S3 Table). The specific function of these genes is described in S4 Table.

### Confirmation of gene signatures with outcome

**Fig 4:**
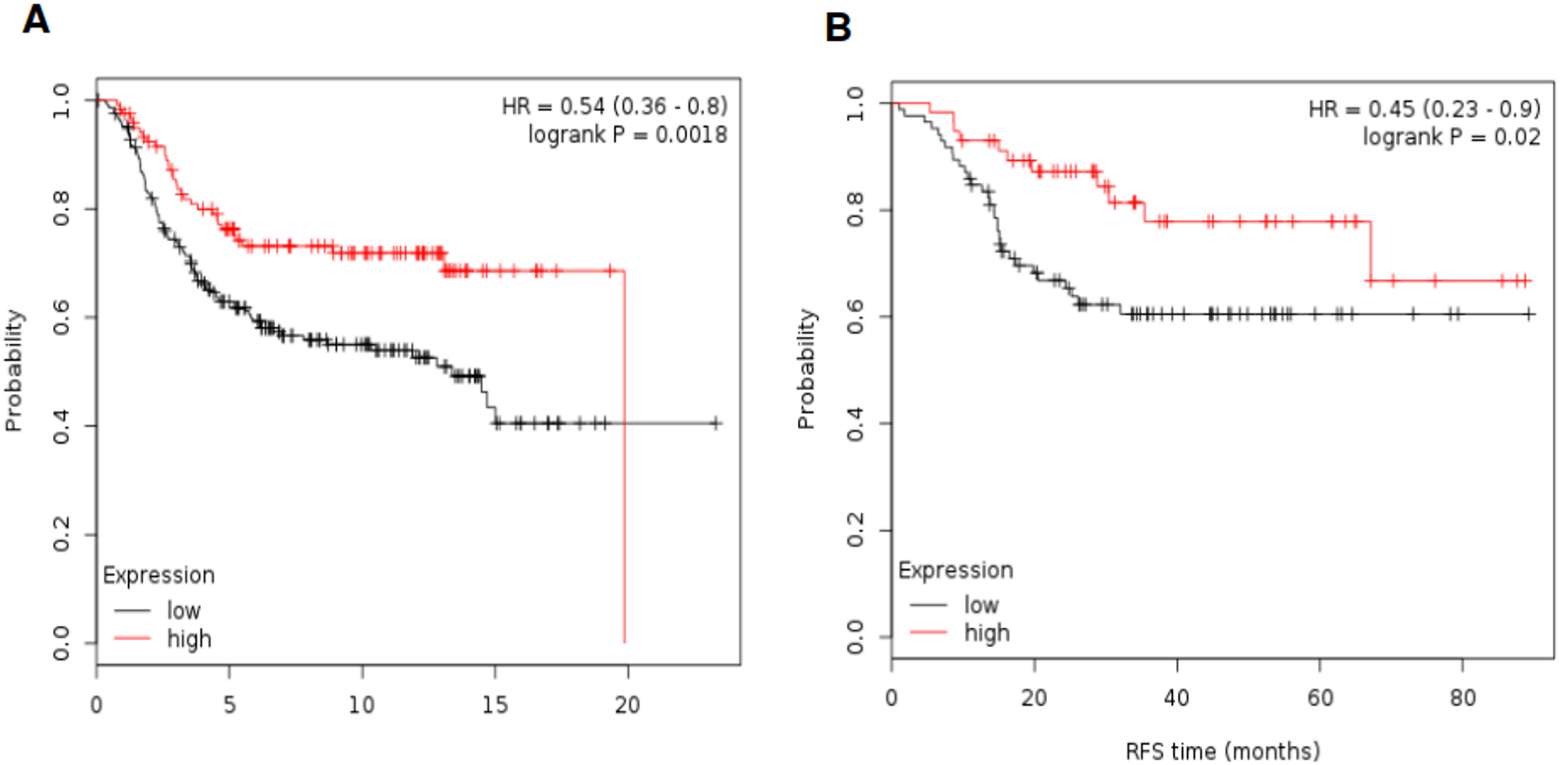
**A.** Association of the combined analyses of HLA-F and HLA-C with OS by using data available in METABRIC. **B**. Association of the combined analyses of HLA-F and HLA-C with RFS by using data available in GSE25066.

To confirm the association of these gene signatures with outcome we used additional public available databases. The METABRIC database [13] contains information on OS in 331 basal-like tumors. Of note the TIGIT gene is not included in this database. Fig 4A shows results associated with outcome. The combination of HLA-C and HLA-F was associated with the better outcome (HR 0.54 [95% CI 0.36–0.80] p = 0.002. Using the GSE25066 dataset [14] that contains RFS data in 142 basal-like patients, the combination of HLA-C and HLA-F showed improved RFS (HR 0,45 [95% CI 0.23–0.90] p = 0.02), confirming the results previously described (Fig 4B). Again this dataset did not include TIGIT.

## Discussion

In our study we intended to explore cell surface transmembrane proteins linked with immunological function and outcome. Cell membrane proteins can easily be used as biomarkers and potentially as therapeutic targets. We found that genes that code for membrane receptors involved in the identification of antigens and regulation of immune response were associated with good prognosis. HLA-C, HLA-F, HLA-G and TIGIT are frequently overexpressed in TNBC compared with normal breast and associated with improved RFS and OS in different datasets.

Using data contained at KM Plotter we observed that HLA-C, HLA-F, HLA-G and TIGIT were associated with good prognosis. Studies in which we used combinations of these genes particularly for HLA-C and HLA-F or HLA-C, HLA-F and TIGIT showed association with good prognosis. We confirmed data for HLA-C and HLA-F combination in two independent datasets, (METABRIC and GSE25066). Unfortunately, TIGIT was not included in either dataset.

Recognition of antigens and activation of T-Cells is a key function in the immune response to cancer [8]. Indeed, hosts that can recognize tumor cells and activate an effector response are those linked with a better outcome. This has been observed when evaluating the association of TIL with outcome, a positive association which has been observed in TNBC [4, 5]. Our data are consistent with this. In addition, the fact that no copy number alterations exist, suggest that upregulation of these genes occurs due to the activation of an immune response.

HLA comprises a set of surface proteins whose main function is to display antigens on the cell surface of antigen presenting cells (APCs) for the recognition by T-cells [8]. TIGIT is a recently discovered immune receptor expressed in T cells and natural killers (NK) [9]. TIGIT has been described as overexpressed in CD8 expressing TILs in melanoma [16].

Although most patients with early stage triple negative breast cancer receive treatment with chemotherapy due to the high risk of relapse of this disease, there is a small number of patients for whom decisions are less clear [17].

This includes patients with small tumors, older patients or those with comorbidities. In this subgroup, additional information such as whether an immune response is anticipated could be valuable. Moreover, the possibility of using agents that may modulate TIGIT activity can also be explored as a therapeutic strategy.

This study has a few limitations. This is an *in silico* and exploratory analysis that needs confirmation in larger studies. In addition, it would be interesting to study whether HLA-C, HLA-F and TIGIT expression is associated with quantitative intra-tumoral TILs.

In conclusion, using three independent datasets, we describe an immunologic signature associated with good outcome in TNBC patients. This signature can be considered as expression of an immunologic response composed of genes associated with the presentation of antigens and the regulation of the immune response. Further studies should be performed to confirm the prognostic value in prospective studies.

## Supporting information

S1 Fig Correlation between cutoff and achieved significance using HLA-F and TIGIT with RFS and OS in basal-like patients. (TIF)

S1 Table List of genes overexpressed between normal breast and basal tumors and classified by functional annotation using DAVID Bioinformatics Resources 6.8. (DOC)

S2 Table Fold changed observed in the identified genes (from GDS2250 dataset) and confirmation of their upregulation using data available in Oncomine, as described in materials and methods. (DOC)

S3 Table Copy number alterations (amplifications/deletions) or mutations in the identified genes using data contained in cBioportal as described in materials and methods. (DOC)

S4 Table Description of identified genes (HLA-C, HLA-F, HLA-G, TIGIT) with their biologic function. (DOC)
