# Supplementary figures and images for: Transcriptomic immunologic signature associated with favorable clinical outcome in basal-like breast tumors
Source: PLoS One. 2017 May 4;12(5):e0175128. doi: 10.1371/journal.pone.0175128 (PMC5417488; doi:10.1371/journal.pone.0175128)

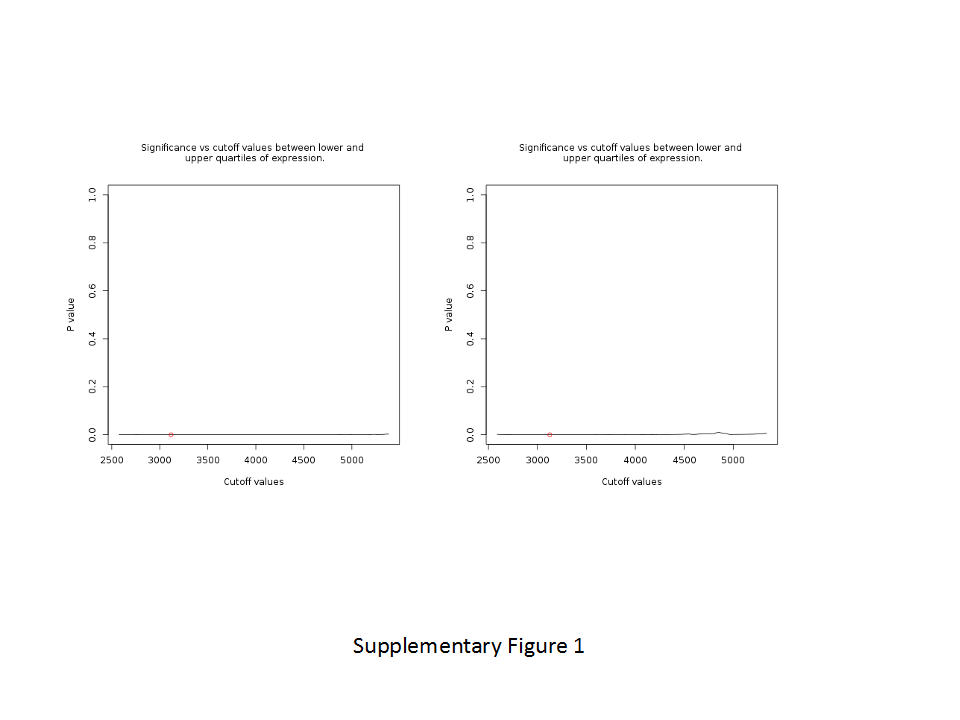

Supplement: S1 Fig — (TIF) [file pone.0175128.s001.tif]
