# Supplementary material for: Transcriptomic immunologic signature associated with favorable clinical outcome in basal-like breast tumors
Source: PLoS One. 2017 May 4;12(5):e0175128. doi: 10.1371/journal.pone.0175128 (PMC5417488; doi:10.1371/journal.pone.0175128)
Supplement: S1 Table — (DOC) [file pone.0175128.s002.doc]

| **Term** | **Genes** | **Adjusted p-value (Benjamini)** | **Count/**  **total list** | **Population hits/total** |
| --- | --- | --- | --- | --- |
|  | | | | |
| **Transmembrane receptor protein**  **tyr- kinase**  **signaling pathway** | ARID5B, ATP6V1C2, ARHGEF9, ELK1, EPHA1, EPHA4, EPHB1, KIT, KANK1, LYN, MET, NCK1, RASGRP1, RRAGD, RASGEF1A, ARHGEF2, SH3KBP1, SHC4, SOX9, TIAM2, YBX1, YES1, ADCY7, ADCYAP1, ago-02, ago-04, ARNT, BMP2, BMP5, BDNF, CDH3, CFL1, CSF2RA, DOK5, DUSP7, EFNA2, EFNA5, EGFR, ESR2, EIF4EBP1, FGF9, FGFR2, FOXC1, FOXC2, GRIN1, GPLD1, HDAC2, IL17RD, JUP, MAPK14, NRG2, NTRK2, NTRK3, NRTN, NCF1, NOG, PIK3C3, PDE1A, PDE1C, PLCE1, PDGFRA, PSMB9, RIPK2, SLC9A6 | 3,40E-01 | 5,90% | 64 / 1419 |
| **Transmembrane transport** | ABCA13, ABCC4, ATP13A3, ATP6V1C2, ATP11C, BCL2A1, CNKSR3, ARHGEF9, FXYD6, NIPAL3, OCA2, S100A1, SEH1L, STEAP3, WNK3, ADD2, ADCY7, AQP4, AQP5, AQP9, CACNA2D1, CSN3, CTSS, CLCA4, CLIC4, CLCN4, CLDN16, CNGB1, CFTR, DAPK1, DMD, FAM26F, GAL, GABRP, GJA5, GJB3, GJC1, GRIA3, GRIN1, GRIK2, KLHL24, LRRC8D, LCN2, MFSD4, NRXN1, NUP153, NUP50, OPRL1, OPRK1, KCNN4, KCNT2, KCNK2, KCNK5, KCNG1, KCNV1, KCNB2, KCNQ4, PRNP, P2RX5, RGN, RYR1, SCARB1, SLC1A2, SLC15A1, SLC16A1, SLC16A10, SLC16A9, SLC2A12, SLC2A6, SLC25A37, SLC26A2, SLC26A9, SLC27A6, SLC34A2, SLC39A4, SLC39A8, SLC43A3, SLC6A2, SLC6A15, SLC7A14, SLC8A1, SLC9A6, SV2B, TRPM6, TRPM8, TRPV4, TMEM38A, TMEM38B, TAP1, TAP2, TTYH1 | 2,50E-01 | 8,30% | 91 / 1419 |
| **Cell**  **adhesion** | PTPLA, BCL11A, CCL5, CXCL13, CX3CL1, CLEC4E, CLEC7A, CD24, CD300A, CD44, CD6, EPHA1, EPHA4, EPHB1, GTPBP4, KIT, KANK1, LRP6, LYPD5, LYN, NCK1, PERP, RAP2B, RASGRP1, RSU1, SATB1, SRCIN1, SOX9, TIGIT, B4GALT1, VTCN1, YES1, ASS1, BMP2, BCAN, BTN2A2, BYSL, CDH12, CDH19, CDH3, CADM4, CLDN1, CLDN16, CLDN6, COL6A6, CNTNAP5, CDK6, CDKN2A, CYTH3, DCHS2, DSC2, DSC3, DSG1, DSG2, DSG3, EFNA5, EGFR, FERMT1, FOXC2, FZD7, GCNT2, GPM6B, GBP1, HAPLN1, HAPLN3, IDO1, ITGA9, ITGB8, ICAM1, ICAM2, IL1RL2, IL32, JUP, LSAMP, HLA-G, MFI2, MSLN, MFGE8, MSN, MUC16, MLLT4, MYO10, MYBPC1, NPHP4, NRXN1, PARVB, PELI1, PPARA, PKP1, PKP2, PDGFRA, PRNP, PRKX, PCDH18, PCDH8, RIPK2, ROPN1B, SCARB1, SFRP1, SCGB1A1, SERPINE2, SAA1, SORBS2, SPOCK2, TRPV4, TNFSF8, TTYH1, UBASH3B, UNC13D, VNN1 | 1,00E-02 | 10,10% | 110 / 1419 |
| **Cell**  **communication** | HTR7, ARL5B, ARL9, AMER2, ARID5B, ATP6V1C2, BCL11A, BARX1, BCL2L14, BCL2A1, BID, BUB1, CCL13, CCL18, CCL20, CCL23, CCL5, CCL8, CXCL1, CXCL10, CXCL11, CXCL13, CXCL5, CX3CL1, CLEC4E, CLEC7A, CD109, CD24, CD300A, CD40, CD44, CD6, CDC42EP1, CDC42EP3, CDC42EP4, CDC42EP5, CNKSR3, CELF4, CADPS, ARHGEF9, CHAC1, DEK, DEPDC1, DIRAS1, ELK1, EPHA1, EPHA4, EPHB1, FBXO18, GPR12, GPR161, GPR180, GPRC5B, GRK4, GMDS, GLIPR2, IQGAP3, KDELR3, KIT, KANK1, LRP12, LRP6, LYN, MET, MX1, NDRG1, NMT2, NCK1, NDC80, NDRG2, NUF2, NDFIP2, PERP, PPARGC1B, PIM1, RSPO2, RAB40B, RAP2A, RAP2B, RASGRP1, RASD2, RCAN3, RQCD1, RBM15, RALGAPA2, RRAGD, RSU1, RASGEF1A, RASGEF1C, ARHGAP22, ARHGAP28, ARHGAP42, ARHGEF2, SKP2, S100A1, S100B, SEH1L, STAC, SH3KBP1 SHC4, SIX3, SRPK1, SOX10, SOX11, SOX8, SOX9, TIAM2, TEAD2, TEAD4, TRAF1, TIFA, TTK, U2AF1, B4GALT1, VANGL2, WIPF3, WNK3, WIF1, WWTR1, WTIP, YBX1, YBX3, YES1, YOD1, ZIC1, ASPM, ATF6B, ACSL5, ACOT11, ADORA2B, ADCY7, ADCYAP1, GPR125, GPR113, GPR64, APBA2, AMOTL1, ago-02, ago-04, ARNT, AURKB, AMBRA1, BMP2, BMP5, BDNF, BCAN, CDH3, CALML5, CHST3, CA8, CTSC, CTSS, CTSV, CDC20, CDC5L, CRABP1, CNTLN, CENPA, CENPF, CENPL, CHI3L1, CHRM1, CHRM3, CHAF1B, CFL1, CSF2RA, CNKSR2, CRYAB, CNGB1, CCNE1, CCNK, CCNY, CDK6, CDKN2A, CDKN2B, CBS, CFTR, CYP26B1, CYP7B1, CYTH3, DAPK1, DNER, DSC2, DSG2, DCBLD2, DLGAP1, DLX5, DOK5, DPF3, DAPP1, DUOX1, DUSP7, DMD, EFNA2, EFNA5, EGFR, ESR2, EIF4EBP1, FAM126A, FAM83D, FGF9, FGFR2, FOLR1, FOXC1, FOXC2, FOXM1, FMNL2, FZD7, FZD9, GAL, GABRP, GABBR2, GJA5, GJB3, GJB5, GJB6, GJC1, GCNT2, GAD2, GRIA3, GRIN1, GRIK2, GRM2, GLS, GSTP1, AGPAT9, GPM6B, GPLD1, GRHL3, GZMB, GREM2, GNB4, GBP1, HRK, HNF4G, HEY2, HMGB2, HDAC2, HDAC9, HAPLN1, IMPA2, ITM2C, ITGA9, ITGB8, ICAM1, ICAM2, IFNAR2, IFIH1, IFI16, IL1RL2, IL1R2, IL12RB2, IL17RD, IL22, IL27RA, JUP, KPNA2, KPNA4, KLHL24, KRT15, LATS2, LGR6, LCN2, LPL, MARCO, HLA-C, HLA-F, HLA-G, HLA-DOB, MGAT3, MAML2, MED30, MESP1, MID1, MAPK14, KIAA1804, MRAS, MSH2, MLLT4, MYO10, NPR3, NPHP4, NRG2, NRXN1, NMU, NTRK2, NTRK3, NRTN, NCF1, NOG, NR2F2, NUP153, NUP50, OR5I1, OPRL1, OPRK1, OXGR1, PAX6, PRRX2, PTCH1, PTCHD1, PELI1, PADI2, PPARA, PMAIP1, PIK3C3, PDE1A, PDE1C, PDE9A, PFKP, PLCE1, PLCH1, PLCL2, PKP1, PKP2, PDGFRA, PLEKHF1, PLEKHG1, PLEKHG4B, PLEKHB1, PLK1, KCNN4, KCTD16, KCNK2, KCNG1, KCNV1, KCNB2, KCNQ4, PRAME, PRICKLE1, PRNP, PML, POMC, PTGFR, PTGIS, PTGS2, PSMB9, PDIA6, PRKD3, PPP1R14A, PPP1R2, PPM1L, PTPN5, PCDH8, P2RX5, RIPK2, RGN, RIMS3, RGS20, RGS8, RCAN1, RRAS2, RGMA, RBP1, RDH10, ROPN1, ROPN1B, RNF175, RYR1, SCARA3, SCARB1, SOSTDC1, SECTM1, SFRP1, SCGB1A1, SERPINE2, SAA1, SESN3, SLC1A2, SLC16A1, SLC39A4, SLC6A2, SLC8A1, SLC9A6, SORBS2, SPOCK2, S1PR5, STMN1, STAR, SYNGR1, SNAP91, TNKS, TSPAN33, TSPAN6, TLR1, TLR6, TLK1, TCF7L1, TFDP1, TRPV4, TSPO, TMOD2, TULP4, TNFRSF10D, TNFSF8, TP53BP2, UCHL1, UCHL5, GABBR1, UBASH3B, AKT3, MYC, VNN1, WNT6, WNT9A | 6,80E-03 | 37,20% | 405 / 1419 |
| **Cell**  **differentiation** | PTPLA, ADAM18, ALX1, ARID5B, ATP11C, BCL11A, BARX1, CCL5, CXCL10, CX3CL1, CLEC4E, CEBPG, CD109, CD24, CMTM7, CELF4, CHAC1, DZIP1, ELF5, ELK1, EPHA1, EPHA4, EPHB1, EHF, ETV6, ETV7, FEZF2, GPRC5B, GLIPR2, GTPBP4, H1FOO, HORMAD1, IQCG, KIT, KANK1, KLF13, KLF15, KLF5, LRP6, LMO4, LYN, MET, MICALL1, MOV10L1, NDRG1, NCK1, NDRG2, OCA2, PLAGL1, PPARGC1B, QKI, RSPO2, RAP2A, RAP2B, RASGRP1, RBM15, RASGEF1A, ARHGAP22, ARHGEF2, S100B, SATB1, SHC4, SIX3, SRCIN1, SRPK1, SOX10, SOX11, SOX6, SOX8, SOX9, SAP30, SPIB, TLX1, TAF4B, TEAD2, UGT8, B4GALT1, VANGL2, WIPF3, WIF1, WWTR1, XK, YBX1, YES1, ZIC1, ASPM, ACTL8, ADCYAP1, ASF1A, APOA5, ARNT, AMBRA1, BMP2, BMP5, BDNF, BYSL, CDH3, CALU, CHST3, CBR1, CTSV, CDC20, CENPF, CLIC4, CHODL, CFL1, COL9A3, COL27A1, CSF2RA, COBL, CRYAB, CNGB1, CCNE1, CDK6, CDKN2A, CDKN2B, CSRP2, CFTR, CYP26B1, DNER, DLX5, DLX6, DUSP7, DMD, EN1, EFNA2, EFNA5, EGFR, ESR2, FAM83D, FABP7, FGF9, FGFR2, FAM101A, FOLR1, FOXC1, FOXC2, FOXD3, FOXG1, FOXM1, FOXP4, FZD7, FZD9, GAL, GJB5, GJC1, GCNT2, GRIN1, GSTA1, GSTP1, GDPD5, GPM6B, GPLD1, GBP1, HEY2, HMGB2, HDAC2, HDAC9, HOXA2, ID4, ITM2C, ITGA9, ICAM1, IFI16, IL1RL2, IL17RD, IL34, KRT14, KRT16, LATS2, LPL, LOR, HLA-G, MED30, MFI2, MESP1, MAP2, MAPK14, MSN, MURC, MBNL1, MBNL3, MSH2, MYRF, MLLT4, MYO10, NPHP4, NRG2, NRXN1, NTRK2, NTRK3, NRTN, NOG, NFIB, NFIX, NR2F2, NFYA, OSR1, PAX6, PTCH1, PCNT, PPARA, PHGDH, PLCL2, PKP2, PDGFRA, PHLDA1, PRAME, PRICKLE1, PRNP, PROM1, PML, PTGS2, PSMB9, PRKX, PTPN5, PURB, RIPK2, RGS20, RRAS2, RGMA, RDH10, RYR1, SGCB, SGCZ, SLFN5, SOSTDC1, SFRP1, SERPINE2, SIM1, SIM2, SLC8A1, SLC9A6, SORBS2, S1PR5, STMN1, STAR, ST14, SUV39H2, TYMS, TOP2A, TCF7L1, TFCP2L1, TRPV4, TSPO, TMOD1, TMOD2, TNFSF8, UCHL1, GABBR1, UBASH3B, USP42, UNC13D, MYC, VNN1, WNT6, WNT9A, ZMYND15, ZBTB18, ZBTB7A, ZNF280B, ZNF503, ZNF521, ZPBP | 2,00E-03 | 24,20% | 264 / 1419 |

Supplementary Table 1
