# Supplementary material for: Transcriptomic immunologic signature associated with favorable clinical outcome in basal-like breast tumors
Source: PLoS One. 2017 May 4;12(5):e0175128. doi: 10.1371/journal.pone.0175128 (PMC5417488; doi:10.1371/journal.pone.0175128)
Supplement: S2 Table — (DOC) [file pone.0175128.s003.doc]

| **Gene Name** | **Fold Change** | **P-value** | **FC**  **Oncomine** | **P-value Oncomine** |
| --- | --- | --- | --- | --- |
| CD300A, CD300a molecule | 2,41 | 3,49E-02 | 1,492 | 1,86E-07 |
| CD6, CD6 molecule | 2,1 | 2,47E-02 | 1,898 | 2,07E-08 |
| DAPP1, dual adaptor of phosphotyrosine and  3-phosphoinositides | 2,01 | 1,73E-02 | 2,177 | 3,46E-09 |
| HLA-C, major histocompatibility complex, class I, C | 2,31 | 4,89E-02 | 1,6 | 3,72E-08 |
| HLA-F, major histocompatibility complex, class I, F | 2,08 | 1,43E-02 | 1,393 | 3,90E-06 |
| HLA-G, major histocompatibility complex, class I, G | 2,3 | 2,89E-02 | 1,517 | 6,04E-08 |
| TLR6, toll-like receptor 6 | 2,89 | 6,59E-04 | 1,271 | 5,36E-04 |
| CSF2RA, colony stimulating factor 2 receptor, alpha, low-affinity (granulocyte-macrophage) | 2,56 | 3,27E-02 | 1,281 | 0,009 |
| TIGIT, T cell immunoreceptor with Ig and  ITIM domains | 2,26 | 4,91E-02 | 2,831 | 8,81E-19 |
| BTN2A2, butyrophilin, subfamily 2, member A2 | 2,76 | 6,92E-03 | 1,254 | 0,008 |

Supplementary Table 2
