# Supplementary material for: Transcriptomic immunologic signature associated with favorable clinical outcome in basal-like breast tumors
Source: PLoS One. 2017 May 4;12(5):e0175128. doi: 10.1371/journal.pone.0175128 (PMC5417488; doi:10.1371/journal.pone.0175128)
Supplement: S3 Table — (DOC) [file pone.0175128.s004.doc]

| **816 Breast Invasive Carcinoma Samples** | | | | |
| --- | --- | --- | --- | --- |
| **Gene Name** | **Amplification** | **Deletion** | **Mutation** | **Multiple alterations** |
| HLA-C, major histocompatibility complex, class I, C | - | - | 0,60% | - |
| HLA-F, major histocompatibility complex, class I, F | - | - | 0,60% | - |
| HLA-G, major histocompatibility complex, class I, G | - | - | 0,20% | - |
| TIGIT, T cell immunoreceptor with Ig and ITIM domains | 1,30% | - | 0,20% | - |

Supplementary Table 3
