# Supplementary material for: Transcriptomic immunologic signature associated with favorable clinical outcome in basal-like breast tumors
Source: PLoS One. 2017 May 4;12(5):e0175128. doi: 10.1371/journal.pone.0175128 (PMC5417488; doi:10.1371/journal.pone.0175128)
Supplement: S4 Table — (DOC) [file pone.0175128.s005.doc]

| **Gene** | **Function** |
| --- | --- |
| HLA-C, major histocompatibility complex, class I, C | Involved in the presentation of foreign antigens to the immune system |
| HLA-F, major histocompatibility complex, class I, F | Involved in the presentation of foreign antigens to the immune system |
| HLA-G, major histocompatibility complex, class I, G | Involved in the presentation of foreign antigens to the immune system. Plays a role in maternal tolerance of the fetus by mediating protection from the deleterious effects of natural killer cells, cytotoxic T-lymphocytes, macrophages and mononuclear cells. |
| TIGIT, T cell immunoreceptor  with Ig and ITIM domains | Binds with high affinity to the poliovirus receptor (PVR) which causes increased secretion of IL10 and decreased secretion of IL12B and suppresses T-cell activation by promoting the generation of mature immunoregulatory dendritic cells |

Supplementary Table 4
